# Research on a Bearing Fault Enhancement Diagnosis Method with Convolutional Neural Network Based on Adaptive Stochastic Resonance

**DOI:** 10.3390/s22228730

**Published:** 2022-11-11

**Authors:** Chen Wang, Zijian Qiao, Zhangjun Huang, Junchen Xu, Shitong Fang, Cailiang Zhang, Jinjun Liu, Ronghua Zhu, Zhihui Lai

**Affiliations:** 1 Shenzhen Key Laboratory of High Performance Nontraditional Manufacturing, College of Mechatronics and Control Engineering, Shenzhen University, Shenzhen 518060, China; 2 Guangdong Key Laboratory of Electromagnetic Control and Intelligent Robots, College of Mechatronics and Control Engineering, Shenzhen University, Shenzhen 518060, China; 3 School of Mechanical Engineering and Mechanics, Ningbo University, Ningbo 315211, China; 4 Ocean College, Zhejiang University, Zhoushan 316021, China; 5 School of Control and Mechanical Engineering, Tianjin Chengjian University, Tianjin 300384, China

**Keywords:** bearing fault diagnosis, stochastic resonance, optimization algorithm, convolutional neural network

## Abstract

As a powerful feature extraction tool, a convolutional neural network (CNN) has strong adaptability for big data applications such as bearing fault diagnosis, whereas the classification performance is limited when the quality of raw signals is poor. In this paper, stochastic resonance (SR), which provides an advanced feature enhancement approach for weak signals with strong background noise, is introduced as a data pre-processing method for the CNN to improve its classification performance. First, a multiparameter adjusting bistable Duffing system that can achieve SR under large-parameter weak signals is introduced. A hybrid optimization algorithm (HOA) combining the genetic algorithm (GA) and the simulated annealing (SA) is proposed to adaptively obtain the optimized parameters and output signal-to-noise ratio (SNR) of the Duffing system. Therefore, the data optimization based on the multiparameter-adjusting SR of Duffing system can be realized. An SR-based mapping method is further proposed to convert the outputs of the Duffing system into grey images, which can be further processed by a normal CNN with batch normalization (BN) layers and dropout layers. After verifying the feasibility of the HOA in multiparameter optimization of the Duffing system, the bearing fault data set from the CWRU bearing data center was processed by the proposed fault enhancement classification and identification method. The research showed that the weak features of the bearing signals could be enhanced significantly through the adaptive multiparameter optimization of SR, and classification accuracies for 10 categories of bearing signals could achieve 100% and those for 20 categories could achieve more than 96.9%, which is better than other methods. The influences of the population number on the classification accuracies and calculation time were further studied, and the feature map and network visualization are presented. It was demonstrated that the proposed method can realize high-performance fault enhancement classification and identification.

## 1. Introduction

Modern mechanical equipment develops towards complexity and automation; hence, minor malfunctions of parts may bring serious chain reactions. Rolling bearings, as a type of important component of rotating machinery, are widely used in wind turbines, compressors, high-speed railways, and other modern mechanical equipment. Bearing failure may cause the failure of the whole equipment, resulting in significant economic loss and even casualties. Therefore, it is necessary to monitor the working condition of rolling bearings and diagnose bearing faults in time to prevent potential accidents, which can be realized by intelligent fault diagnosis methods widely studied in recent years [1,2].

The framework for an intelligent fault diagnosis method can be divided into three parts [3]: signal acquisition, fault feature extraction, and fault classification. The objects of the signal acquisition include many kinds of signals, such as vibration signals [4], acoustic signals [5] and electrical signals [6], among which the vibration signals are most focused as they contain rich essential information of mechanical faults. Common signal processing methods for feature extraction features include short Fourier transform (STFT) [7], wavelet transform (WT) [8], empirical mode decomposition (EMD) [9], principal component analysis (PCA) [10] and stochastic resonance (SR) [11,12], etc. Fault classification is used to determine the health conditions of mechanical equipment. Common fault classification methods include deep learning, support vector machine (SVM) [13], and the K-nearest neighbor (KNN) algorithm [14]. The selections of the fault feature extraction method and the fault classification method are key to establishing a vibration-signal-based fault diagnosis method.

In practical applications, the fault features in the vibration signals are always weak when the fault is in its early stage or the faulty part operates in a terrible working environment. The weak features are difficult to extract through a normal feature extraction method, thus limiting the weak fault diagnosis performance of the corresponding intelligent fault diagnosis methods. Therefore, a feature extraction method with the capability of weak-signal enhancement should be selected in these cases. The stochastic resonance (SR) -based methods are a type of weak feature extraction method that can obtain a higher signal-to-noise ratio (SNR) compared to other traditional feature extraction methods [15]. Stochastic resonance is a type of commonly seen nonlinear phenomenon, which was first proposed by Benzi in the 1980s to describe the periodicity associated with the ice age of the earth in climatology [16]; it occurs under the cooperation of input weak signal, noise and a nonlinear system, which enables the weak signal to increase its amplitude by absorbing energy from noise. Hence, this interesting phenomenon is widely applied in physics and engineering research [17], such as energy harvesting [18] and weak-signal detection in bearing fault diagnosis [19,20]. Previous research shows that the weak-signal enhancement performance of the SR system is significantly influenced by the nonlinear system, which involves monostable systems [21,22], bistable systems [23,24,25], tri-stable systems [26] and multi-stable systems [27]. Among these nonlinear systems, bistable systems such as the Duffing system are widely studied due to their advanced weak-signal detection performance [28].

However, SR systems need to meet the small-parameter conditions [29] (the frequency and amplitude of weak-signal should be within small-parameter ranges) due to the adiabatic approximation theory, which cannot be satisfied for most practical vibration signals. To solve this issue, a multiparameter adjusting SR system has been proposed [30] by introducing an amplitude transformation coefficient and a frequency transformation coefficient, which are used to transform the amplitude and frequency of the signals to appropriate small-parameter ranges. Therefore, SR for larger-parameter signals can be achieved. In order to realize the SR output of the multiparameter adjusting system, the optimal parameters should be obtained, which can be realized by adaptive multiparameter optimization methods [31], such as the particle swarm optimization (PSO) [32], genetic algorithm (GA) [33] and simulated annealing (SA) algorithm [34], etc.; however, these algorithms all have their limitations in achieving optimal SR outputs. For example, the GA has a powerful global search capability but it is easy to trap in the local optimum [35], while SA can escape from the local optimum but has a slow optimization speed [36]. Therefore, the combination of some of these algorithms can improve their optimization performance, which has been studied in some literature [37].

As for the fault classification method, deep learning technology has achieved great success in many fields in recent years due to its powerful feature extraction and classification capabilities [38,39]. The deep learning technologies include deep neural network (DNN) [40], recurrent neural network (RNN) [41] and convolutional neural network (CNN) [42], etc. They can extract features from input automatically by building multiple neural layers and make predictions accordingly, which can also be used in many application fields including the fault diagnosis of mechanical equipment. In the past years, deep learning has been used as an advanced classification tool, which can effectively classify the signals obtained from traditional feature extraction methods [43]. In this paper, the CNN, which is one of the main types of deep learning technology and has been applied widely in fault classification, was selected as the fault classification method. However, it was noted that its classification accuracy was not high enough when the quality of the raw data was poor [44], such as the weak fault signals. Therefore, it is necessary to pre-process the raw data using a weak-fault feature extraction method to extract useful features, which can be further classified by the CNN.

In this work, a fault enhancement classification method combining the adaptive SR, which utilizes a hybrid optimization method combining the SA and GA as the weak-fault feature extraction method and a normal CNN as the fault classification method, is proposed for high-performance bearing weak-fault diagnosis. This paper is organized as follows. The multiparameter adjusting bistable Duffing system, the hybrid optimization method and a mapping method are introduced in Section 2. In Section 3, the normal CNN is presented by appropriately selecting its parameters. A series of simulations and experiments are conducted in Section 4 to verify the proposed signal pre-processing and fault diagnosis methods. Conclusions are drawn in Section 5.

## 2. Data Preprocessing Based on Adaptive Multiparameter Optimization of SR

In this section, the classical bistable Duffing system we investigated previously is introduced as a data preprocessing model for the further classification algorithm. A hybrid intelligent optimization algorithm combining genetic algorithm (GA) and simulated annealing (SA) was used to achieve stochastic resonance (SR) in this system and obtain the optimal parameters. To make the SR outputs able to be processed by the CNN for classification, a mapping method based on a noise intensity sequence is further proposed to convert the time-domain output of the Duffing system into an image that can be further processed in classification.

### 2.1. Introduction of the Bistable Duffing System That Can Achieve SR

The bistable Duffing system, which is a typical form of nonlinear system that can achieve SR, can be described as [20]:

(1) $$\ddot{x}(t)+k\dot{x}(t)-ax(t)+bx^{3}(t)=s(t)+n(t)$$

where k denotes the damping ratio; a and b are system parameters deciding the potential function of the system; $s(t)=A\cos(2\pi f_{0}t)$ indicates a harmonic characteristic signal with amplitude A and frequency $f_{0}$; $n(t)=\sqrt{2D}\xi (t)$ indicates a Gaussian white noise with noise intensity D, where ξ(t) is a zero-mean and unit-variance Gaussian white noise. In this system, sn(t)=s(t)+n(t) is defined as the input signal, and x(t) is the output signal, which can be obtained by solving Equation (1) numerically.

**Figure 1:**
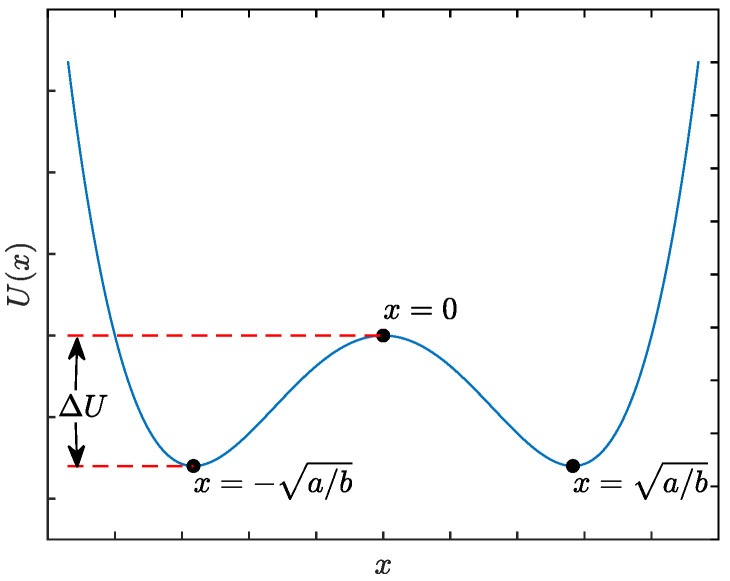
Bistable potential function without input.

In Equation (1), the term of $dU(x)/dx=-ax+bx^{3}$ can be understood as the tangential force of a bistable potential field given by $U(x)=-ax^{2}/2+bx^{4}/4$, which has two stable equilibrium points at $x=\pm \sqrt{a/b}$ and one unstable equilibrium point at x=0, as shown in Figure 1. This shows that a potential barrier with a height of $\Delta U=a^{2}/(4b)$ separates two symmetrical potential wells, showing why the Duffing system is bistable. Moreover, the output x(t) of Equation (1) can be understood as the trajectory of a unit-mass Brownian particle moving in the potential field U(x), which is suffered from the damping force $-k\dot{x}(t)$ and the external excitation sn(t) as well. Stochastic resonance indicates an optimal matching result between the signal, noise, and a nonlinear system. When SR occurs, the particle can get energy from noise and cross the barrier regularly even though the amplitude of the signal is relatively low, thus enhancing the weak features of the weak signal. Hence, SR provides a feasible way to extract the features of the input signal from the enhanced output signal, especially under weak-signal conditions.

Previous research results show that due to the adiabatic approximation theory, the bistable Duffing system can only achieve SR under small-parameter conditions, i.e., the amplitude, frequency and noise intensity should be small [30]; however, most of the practical engineering signals do not satisfy this small-parameter requirement. Hence, to enhance the signal features of such large-parameter signals, an improved multiparameter-adjusting SR model based on the bistable Duffing system was proposed by the authors previously [45]. By introducing two adjusted parameters ε and R, this model can be written as:

(2) $$\ddot{x}(t^{\prime })+k\dot{x}(t^{\prime })-ax(t^{\prime })+bx{\left(t^{\prime }\right)}^{3}=\varepsilon \cdot sn(t^{\prime })$$

where ε is the amplitude transform coefficient used to transform the amplitude of the input signal to an appropriate range, and R is the scale-transformation coefficient used to transform the time scale of the input signal from t to $t^{\prime }=Rt$. The scale transformation can be simply realized by applying a time step of $\Delta t^{\prime }=R/f_{s}$ instead of $\Delta t=1/f_{s}$ in numerical calculation, where $f_{s}$ denotes the sampling frequency of the system. Therefore, the frequency of the characteristic signal ($f_{0}$) can be regarded as $f_{0}/R$ in the numerical calculation. The large frequency of the input signal can be compressed accordingly in the numerical calculation by setting an appropriate value of R.

The output signal-to-noise ratio (SNR) of the Duffing system can be regarded as an objective optimization function to decide whether the system achieves SR. The output SNR of the system is defined as:

(3) $$\left\{ \begin{array}{l} {\mathrm{SNR}}_{\mathrm{in}}=10\log_{10}\left(\frac{{\mathrm{Am}}_{\mathrm{in}}^{2}}{\sum {\left[\mathrm{SN}(f)\right]}^{2}-{\mathrm{Am}}_{\mathrm{in}}^{2}}\right)\\ {\mathrm{SNR}}_{\mathrm{out}}=10\log_{10}\left(\frac{{\mathrm{Am}}_{\mathrm{out}}^{2}}{\sum {\left[X(f)\right]}^{2}-{\mathrm{Am}}_{\mathrm{out}}^{2}}\right) \end{array}\right.$$

where SN(f) represents the single-side spectrum of input sn(t), and X(f) represents the single-side spectrum of the system output x(t). Moreover, ${\mathrm{Am}}_{\mathrm{in}}=\mathrm{SN}(f_{0})$ and ${\mathrm{Am}}_{\mathrm{out}}=X(f_{0})$ indicates the amplitudes of the system input signal and output signal at the characteristic frequency $f=f_{0}$.

### 2.2. Hybrid Optimization Algorithm Combining GA and SA

To achieve SR adaptively in a bistable Duffing system under an input signal with fixed parameters, an optimization algorithm is needed to obtain a group of appropriate system parameters (a, b), damping ratio (k) and adjusted parameters (ε, R) that match the fixed input signal. Among various optimization algorithms, the genetic algorithm (GA) is an effective intelligent optimization algorithm when the objective optimization function is not differentiable, and it can obtain a local optimization value greater than 90% of the global optimization one in a short time [35]. In the GA, every individual represents a solution, and its principle is to obtain the optimal population by selecting the parents to do cross and mutation according to the fitness function.

However, the optimal results obtained from the GA are local optimization solutions, which could become better for the objective optimization function (such as the output SNR of the Duffing system). This can be obtained by adopting the simulated annealing (SA), which is another important optimization algorithm proposed by Metropolis et al. in 1953 based on the solid annealing process in physics. The SA can accept a solution worse than the current one with a certain probability, resulting in a capacity of jumping out of the local optimal solution and reaching the global optimal solution. The probability of accepting a new solution in the Metropolis criterion in this paper is defined as:

(4) $$P=\{\begin{array}{cccc} 1 & , & & E_{t+1}>E_{t}\\ e^{\frac{E_{t+1}-E_{t}}{kT}} & , & & E_{t+1}\leq E_{t} \end{array}$$

where $E_{t+1}$ and $E_{t}$ are the new condition and temporary optimal condition, respectively, where T is the current updated temperature, and k is a Boltzmann constant set as 1 in this work [34]. The new condition is undoubtedly accepted as the updated temporary optimal condition when $E_{t+1}>E_{t}$; while when $E_{t+1}\leq E_{t}$, the new condition can be also accepted if the acceptance probability P is greater than a random number between [0,1], thus finally obtaining a satisfactory optimization result. The main disadvantage of the SA is its slow optimization speed, which is not satisfied in practical conditions such as the adaptive multiparameter optimization of SR for big data.

Therefore, a hybrid optimization algorithm (HOA) based on both the GA and the SA combining their advantages was utilized in this work for high-performance multiparameter optimization of SR. In the HOA, the Metropolis criterion of the SA was added in the parents’ selection of cross and mutate stages based on the GA framework. Hence, the HOA can get a better optimization solution through the capacity of the SA that jumps out of local optimization in a short time. As a result, we can get an acceptable SNR for the Duffing system in a short time using the HOA.

### 2.3. Data Optimization Based on Multiparameter-Adjusting SR of Duffing System

According to previous analyses, the proposed HOA provides an effective approach to achieving multiparameter-adjusting SR in a Duffing system, thus improving the quality of the input raw signal, and enhancing its SNR. The relevant data optimization method is presented in this subsection.

**Figure 2:**
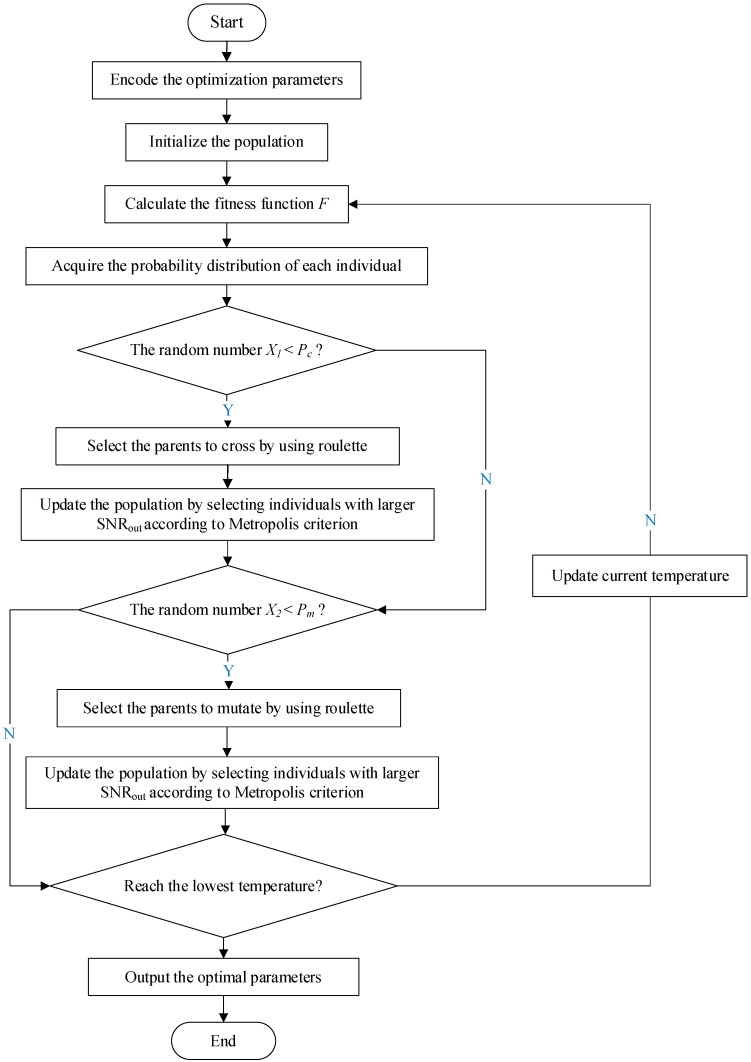
Flowchart of the optimization process to achieve multiparameter-adjusting SR of the Duffing system using the hybrid optimization algorithm.

It is noted that the fourth-order Runge–Kutta algorithm is adopted in this work to solve the Duffing system. The HOA used in this paper adopts a binary encoding format, and each parameter consists of 15-bit binary numbers to guarantee sufficient resolution. Moreover, the value of R is pre-set as an appropriate value to ensure that the calculation results will not overflow. Hence, the optimization parameter dimension of the Duffing system is 4 (k, a, b, and ε) in the optimization, and the flowchart of the optimization process to achieve multiparameter-adjusting SR of the Duffing system using the hybrid optimization algorithm is shown in Figure 2.

In order to use roulette to select the crossed parents and mutation parents of the HOA (see Figure 2), a fitness function F is defined:

(5) $$F=E_{j}^{i}-E_{j}+\delta$$

where j=1,2,3,…,L, and L is the maximum number of iterations; $E_{j}$ denotes the minimum value of the $\mathrm{SN}R_{\mathrm{out}}$ in the whole population in the $j^{th}$ iteration, and $E_{j}^{i}$ denotes the value of the $\mathrm{SN}R_{\mathrm{out}}$ of the $i^{th}$ individual in the $j^{th}$ iteration. It is noted that δ with a quite small value is used in Equation (5) to avoid the fitness function equaling to 0 (δ=0.001 is set in this work), thus, the optimization parameters in terms of minimum $\mathrm{SN}R_{\mathrm{out}}$ can be abandoned. Hence, the value of the fitness function F is always positive. Other parameters in the HOA were set as follows: population number PN=50, cross probability $P_{c}=0.9$, mutation probability $P_{m}=0.9$, initial temperature T=10, minimum temperature $T_{\min}=0.001$ and update weight Δ=0.9. It is necessary to mention that $X_{1}$ and $X_{2}$ represent the random numbers in the cross and mutate stages of GA.

### 2.4. SR-Based Mapping Method with a Noise Intensity Sequence

When using the SR-based methods, engineers are required to have extensive experience, hence, it takes a lot of time and manpower to find out the characteristic frequency of the bearing fault vibration signal. This can be solved by using an intelligent diagnosis method proposed in this work that combines SR with a neural network classifier. For this purpose, the SR output of the Duffing system should be converted into a grey image, which can be further used for feature extraction and fault classification by the neural network. The process of the mapping method is as follows.

First, M continuous time domain points are intercepted from a raw signal to form a new signal $s_{1}(t)$, which is always a noisy signal. To produce more feature information in one image, a sequence of noise $n_{k}(t)$, whose noise densities are given by

(6) $$D_{k}=0.04\times k$$

is further added to the signal $s_{1}(t)$. Therefore, a sequence of input signals can be obtained:

(7) $$sn_{1k}(t)=s_{1}(t)+n_{k}(t)$$

where k=1,2,3,…,m, where m is the pre-set number of the input signals.

Next, by inputting $sn_{1k}(t)$ into the Duffing system of Equation (2), an optimal parameter sets $P_{k}=\left[k_{k},a_{k},b_{k},\varepsilon_{k}\right]$ and the output signal $x_{k}(t)$ can be obtained based on the proposed data optimization method. Therefore, the matrix of the output signals $G=\left[x_{1}(t),x_{2}(t),\ldots ,x_{m}(t)\right]$ can be further converted into a visual grey matrix $G_{gray}$ according to:

(8) $$G_{gray}(i,j)=\frac{G(i,j)-g_{\min}}{g_{\max}-g_{\min}}$$

where i=1,2,…,M and j=1,2,…,m; $g_{\max}$ and $g_{\min}$ are the maximum and minimum values of G(i,j). It is noted that M=512 and m=128 are pre-set in this work.

Hence, for each detected signal, a grey image can be obtained by adding a group of noises with different intensities into the input signal and then being processed by the adaptive multiparameter adjusting Duffing system. More detected signals can produce more grey images, which can be further used by the convolutional neural network (CNN) for feature extraction and fault diagnosis.

## 3. Construction of the CNN

In this section, basic knowledge of a CNN is briefly introduced. The network architecture used for fault classification was obtained by modifying the parameters of the conventional visual geometry group (VGG) net architecture to satisfy the resolution of grey images obtained by the proposed mapping method. It is noted that compared to the conventional VGG, the batch normalization (BN) modules are added in the convolution layer and the dropout modules are added in the full connected (FC) layer to enhance the generalization of the VGG in this work.

### 3.1. Brief Introduction of CNN

The architecture of CNN is briefly introduced in this subsection. A CNN consists of some filter stage and one classification stage [46]. The filter stage contains convolutional layers, activation layers, BN layers, and pooling layers.

The convolutional layer convolves the input local region with kernel filters, and the following activation layer generates a feature map. The kernel that extracts the local features keeps the same in each filter, thus reducing the complication of CNN. The convolution process is described as follows:

(9) $$y^{l+1}=x_{j}^{l}*K_{i}^{l+1}+b_{i}^{l+1}$$

where * is a convolutional operator; $K_{i}^{l+1}$ and $b_{i}^{l+1}$ represent the weight and bias of the ith kernel filter from layer l to layer l+1; $x_{j}^{l}$ denotes the jth local region of layer l, $y^{l+1}$ denotes the output of layer l calculated by convolution. Moreover, the padding method is used in convolution to make full use of all the features of the grey images.

In the activation layer, a nonlinear activation function of Rectified Linear Unit (ReLU) is widely used to improve the expression ability of the whole network, which means the functions that can be expressed are more abundant. The ReLU can prevent the occurrence of overfitting by making the output of some neurons to be zero, thus resulting in the sparsity of networks.

A BN layer is further designed to speed up training and convergence of the network and reduce the shift of internal covariance. The pooling layer generally adopts the max-pooling layer, which enhances the generalization of the model by reducing the parameters while retaining the main features.

Moreover, the classification stage is composed of several FC layers. The FC layer is used for enhancing the generation of the model after convolution, and the number of neurons in the output layer denotes the types of bearing health conditions.

### 3.2. Architecture of the Proposed CNN Model

**Figure 3:**
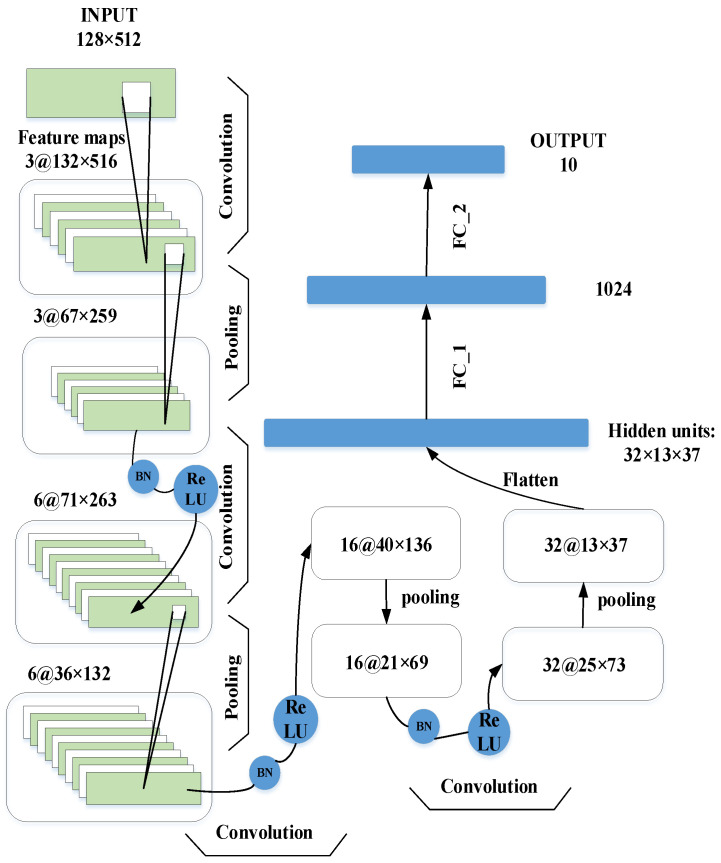
The architecture of the CNN model used in this work.

The whole architecture of the CNN used in this work is shown in Figure 3, which includes convolutional layers, ReLU layers, BN layers, max-pooling layers, and FC layers. The number of convolutional layers depends on the size of the grey image produced by the proposed mapping method. Small convolutional kernels make the networks deeper, which helps to improve the generalization ability of the network, and the size of the convolutional kernel is set as 5×5 accordingly. The BN is implemented after the convolutional layers to accelerate the training process, and the ReLU is utilized in the next layer to prevent the occurrence of overfitting. Max-pooling is used to reduce the parameters of the networks, and the kernel size is set as 2×2. The classification stage includes three FC layers for classification, and the output layer has ten outputs, which represent ten different bearing health conditions.

In the process of training, the number of iterations was set as 300, and an Adam optimizer was utilized to minimize the loss function, where the learning rate was set as Lr=0.001 initially. After every 100 iterations, the learning rate reduced 10 times to get more accurate optimal solutions.

## 4. Verification of the Proposed Method

The proposed method, which can be used for fault classification and fault identification in practical applications, is verified in this section. It is necessary to point out that the computer used for numerical simulations had a CPU of Intel(R) Core (TM) i5-10400 and RAM with 16.00 GB as its main configurations.

For practical noisy fault signals, the characteristic frequency $f_{0}$ of the fault signal is always unknown in advance. Therefore, the characteristic frequency $f_{0}$ should be pre-estimated according to the specific working environment before fault diagnosis, and the objective function $\mathrm{SN}R_{\mathrm{out}}$ for optimization and $SNR_{in}$ for comparison are redefined as follows:

(10) $$\mathrm{SN}R_{\mathrm{out}}=10\log_{10}(\frac{\max{\left\{X(f){|}_{f_{l}\leq f\leq f_{h}}\right\}}^{2}}{{\sum \left[X(f)\right]}^{2}-\max{\left\{X(f){|}_{f_{l}\leq f\leq f_{h}}\right\}}^{2}})$$

(11) $$\mathrm{SN}R_{in}=10\log_{10}(\frac{\max{\left\{\mathrm{SN}(f){|}_{f_{l}\leq f\leq f_{h}}\right\}}^{2}}{{\sum \left[\mathrm{SN}(f)\right]}^{2}-\max{\left\{\mathrm{SN}(f){|}_{f_{l}\leq f\leq f_{h}}\right\}}^{2}})$$

where $f_{l}=f_{0}/R-\Delta f$ and $f_{h}=f_{0}/R+\Delta f$; where $\Delta f=f_{s}/(RM)$ is the adjusted frequency resolution after scale transformation. Consequently, several spectral lines around the pre-estimated frequency are involved to avoid the characteristic spectral line being missing.

### 4.1. Verification of the HOA

In this subsection, the advantage of the HOA is verified by processing a simulated signal with different injected noises. The signal was set as a pure harmonic signal with amplitude A=0.1 and frequency $f_{0}=40$ Hz; the sampling frequency $f_{s}=20000$ Hz and the sampling points N=2000. This signal was injected with noises of different intensities $D_{k}$ ranging from 0.04 to 5.12, and the obtained noisy signals were then input into the multiparameter-adjusting Duffing system shown in Equation (2). Both the HOA and the conventional GA were used to find the optimal $\mathrm{SN}R_{\mathrm{out}}$ for each signal.

**Figure 4:**
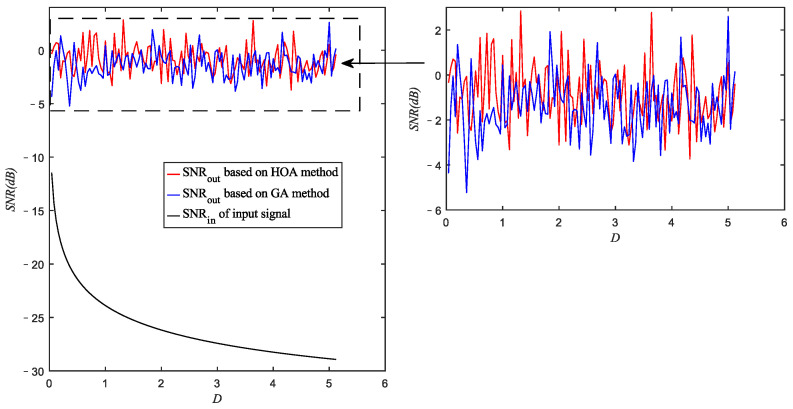
SNR of the optimization results based on HOA and GA with a population number of 500 and SNR of the input signal against noise intensity.

Before optimizations, the value of R should be determined, and the range of each adjustable parameter (k, a, b, ε) should be selected as well. The value of R, which is the scale transformation parameter, was fixed as R=2000 according to the large frequency domain and the large sampling frequency $f_{s}$ to ensure that the calculation results will not overflow in the numerical simulation. Moreover, ε is the amplitude transformation coefficient, whose selected range should be determined according to the amplitude of the input signal. Based on our previous research [30], the value of $\varepsilon \bar{A}$ should be between 0.001 and 0.1, where $\bar{A}=\sum_{f_{1}<f<f_{2}}A(f)/n$ with A(f) the single side spectrum of the input signal and n the number of spectral lines during the frequency range of $\left[f_{1},f_{2}\right]$, which is the manual-selected frequency range involving $f_{0}$. In our simulation, $f_{1}=35$ Hz and $f_{2}=45$ Hz were set. For the given signals with different noises, the values of $\bar{A}$ are within [0.0056,0.2416], hence, the range of [0.2,0.4] was selected for ε to guarantee $0.001<\varepsilon \bar{A}<0.1$. Moreover, the selected ranges of the other three parameters were set artificially as a∈[0.1,5], b∈[0.1,5] and k∈[0.1,5] in optimization. Under different noise intensities, the optimal $\mathrm{SN}R_{\mathrm{out}}$ of the Duffing system obtained from both the HOA and the GA with a population number of 500 are shown in Figure 4. The SNR of input signals with different noise intensities are plotted in this figure as well.

Figure 4 shows that the $\mathrm{SN}R_{\mathrm{in}}$ of input signal presents a decreasing trend as the noise intensity increases, while both the HOA and the GA can achieve a relatively high $\mathrm{SN}R_{\mathrm{out}}$ regardless of the noise intensity, demonstrating the feasibility of the multiparameter optimization algorithms in achieving SR in the Duffing system. Moreover, in most cases (80 of 128) the optimal $\mathrm{SN}R_{\mathrm{out}}$ obtained from the HOA is larger than that obtained from the GA. The advantage of the HOA is it can be also quantitatively concluded that the average value of the optimal $\mathrm{SN}R_{\mathrm{out}}$ obtained from the HOA (−0.8616 dB) is larger than that obtained from the GA (−1.3808 dB). This result indicates that the HOA has a higher possibility to obtain a better local optimization result compared to the GA.

Besides, it is convenient to set a large population number to obtain a local optimization close to the global optimization with a large $\mathrm{SN}R_{\mathrm{out}}$, but it takes a lot of extra time. Whereas, in practical analysis, the time for fault diagnosis is relatively short. Therefore, a smaller population number should be used in practical engineering to achieve acceptable time cost and $\mathrm{SN}R_{\mathrm{out}}$. Its influence on the classification results will be studied in Section 4.2.4. The population number was set as 50 in the following of this section.

### 4.2. Application for Practical Bearing Fault Data Classification

#### 4.2.1. Introduction of the Used Bearing Fault Data Set

**Figure 5:**
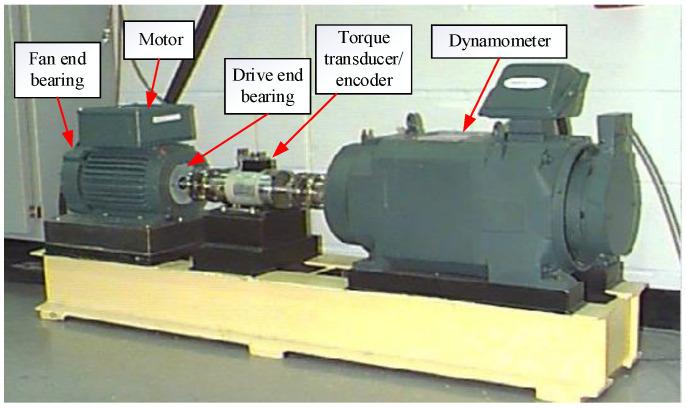
CWRU bearing test rig.

In this subsection, the vibration signals of the rotating bearing from the bearing data center of Case Western Reserve University (CWRU) were processed by the proposed method, thus verifying its feasibility in bearing fault data classification and fault diagnosis. The test rig is shown in Figure 5, which contained a motor with a load of up to 3 hp, a torque transducer or encoder, and a dynamometer.

In the test rig, the test bearings, which were deep groove ball bearings of type 6205-2RS JEM SKF, were used to support the motor shaft. The bearing details are listed in Table 1. In the test, motor bearings were seeded with faults using electro-discharge machining. The diameters of the faults ranged from 0.007 to 0.04 inches, and the faults were separately located at the inner ring, outer ring and rolling element. Faulty bearings were installed onto the test motor, and the vibration data was recorded under the load of 0 to 3 hp (the motor speed was 1797 to 1720 rpm). Therefore, the bearings contained different faults with different health conditions, producing a variety of vibration signals of faulty bearings when they operated.

The bearing data used in the experiment was sampled at the end of the drive with a sampling frequency of 12,000 Hz. As the location of the fault relative to the bearing load area affected the vibration response of the whole motor system, the bearing data at 6 o’clock, 3 o’clock and 12 o’clock directions of the bearing load area were listed, respectively. In this work, the bearing data at 6 o’clock was used for verification.

**Figure 6:**
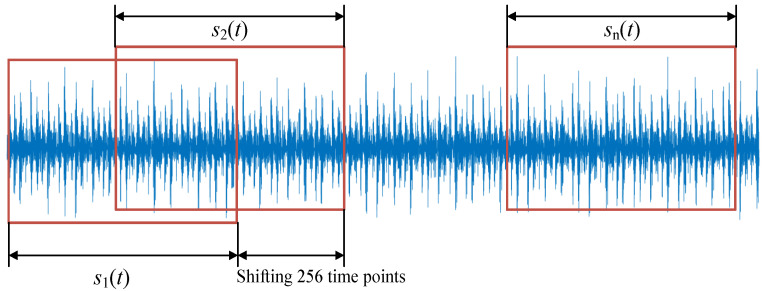
The signal expanding method used in this work.

However, the bearing data set only contains one bearing signal of each fault type, which is not enough for data training. To obtain more fault signals to make the classification results more generalized, each bearing signal was expanded to 400 samples, as shown in Figure 6. The first 512 time domain points of each bearing signal are intercepted from each bearing signal, thus forming a new signal indicated as $s_{1}(t)$. Next, the 257th to 768th time domain points of the bearing signal are intercepted to form a new signal indicated as $s_{2}(t)$. More new signals can be further obtained using the same method. In this work, each bearing signal was expanded to 400 new signals. Moreover, to reduce the amount of computation and save time cost, the parameter set optimized by $s_{1}(t)$ was used for other expanded new signals.

The details of the datasets are shown in Table 2. The datasets contain the signal of the 10 different healthy condition categories under four different loads of 1, 2, 3 and 4 hp, which are represented as datasets A, B, C and D, respectively. Each bearing signal was expanded to 400 samples, among which 320 samples were training samples and 80 samples were testing samples.

#### 4.2.2. Optimization Results

In this subsection, the samples of dataset A are taken as examples to be processed by adaptive optimization of SR. As we mentioned at the beginning of Section 4, the characteristic frequencies of the faulty bearings should be estimated first to calculate the $\mathrm{SN}R_{\mathrm{out}}$. When the system rotated at a constant speed, the characteristic frequency of the bearings can be calculated according to Table 1.

In the optimization processes, the scale transformation parameter was set as R=2000, thus, the frequency resolution Δf at sampling frequency of 12,000 Hz can be obtained as ∼0.0117 Hz. The optimization ranges of other adjustable parameters are pre-set, as shown in Table 3. Note that the characteristic frequency of the normal bearing signals cannot be estimated. As a result, the parameters of the Duffing system with normal bearing signals input cannot be optimized, whereas the normal bearing signals are also processed by the Duffing system with a group of fixed parameters (R=2000, k=0.5, a=b=1, ε=10) to maintain the uniformity compared to other 9 fault bearing signals.

**Figure 7:**
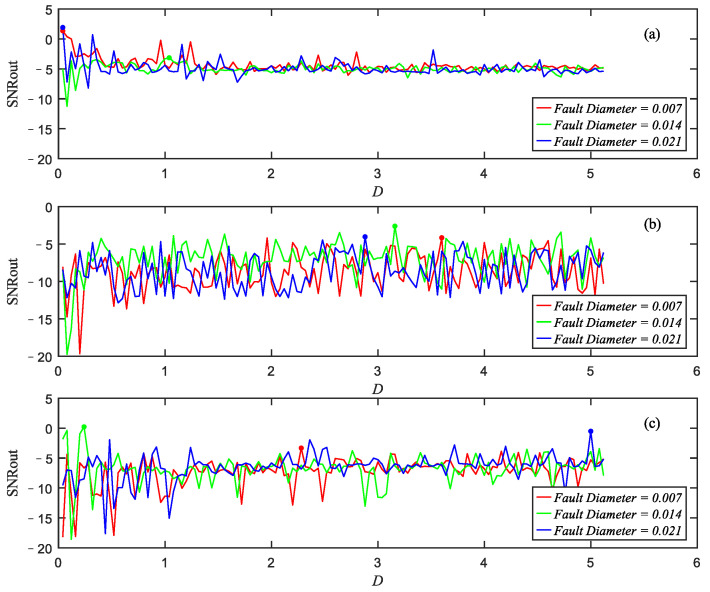
The optimized $\mathrm{SN}R_{\mathrm{out}}$ of the faulty bearing signals with faults of (**a**) inner ring, (**b**) rolling element and (**c**) outer ring against the intensity of injected noise.

Hence, the $\mathrm{SN}R_{\mathrm{out}}$ of the Duffing system with different input signals can be optimized according to the HOA. For example, the values of the optimized $\mathrm{SN}R_{\mathrm{out}}$ for $s_{1}(t)$ of the faulty signals of dataset A against the noise intensity of the injected noise are shown in Figure 7. It is necessary to mention that the $\mathrm{SN}R_{\mathrm{in}}$ curves are not drawn because signal numbers are too small to find precise feature frequency, but the $\mathrm{SN}R_{\mathrm{in}}$ can be obtained by using Equation (11), which are less than −30 dB. Figure 7 shows that all the $\mathrm{SN}R_{\mathrm{out}}$ are more than −20 dB. It can be obtained that the optimized $\mathrm{SN}R_{\mathrm{out}}$ has significant enhancements compared to the $\mathrm{SN}R_{\mathrm{in}}$, demonstrating the feasibly and effectiveness of the HOA in enhancing the weak features of practical signals.

#### 4.2.3. Accuracies of Classification

**Figure 8:**
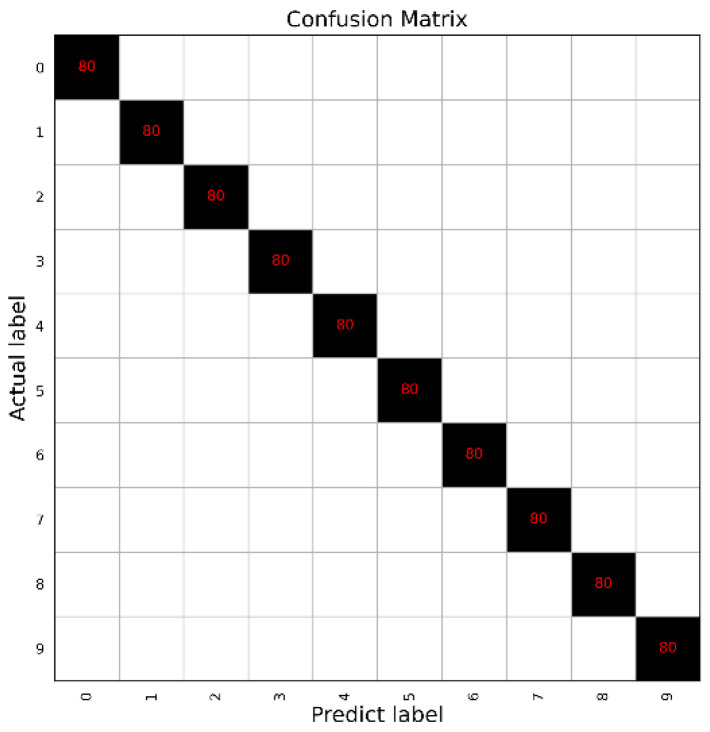
The confusion matrix of dataset A (10 healthy conditions) with 100% accuracy using optimal signals with our CNN architecture.

The classification accuracies of the bearing signals are presented in this subsection. It is noted that the batch size has a significant influence on the coverage speed and classification result. In this work, the batch size was set as 32 to obtain the highest classification accuracy with a relatively high convergence speed. Through simulations, an accuracy of 100% can be obtained in Table 4 for the 10 categories of all datasets presented in Table 2, which is higher than that obtained from other classification methods including SVM, Multilayer Perceptron (MLP), and DNN [47], showing that the proposed method has a good performance in fault classification and feature extraction. Moreover, Figure 8 shows the confusion matrix of dataset A, which clearly shows that each label was classified well.

**Figure 9:**
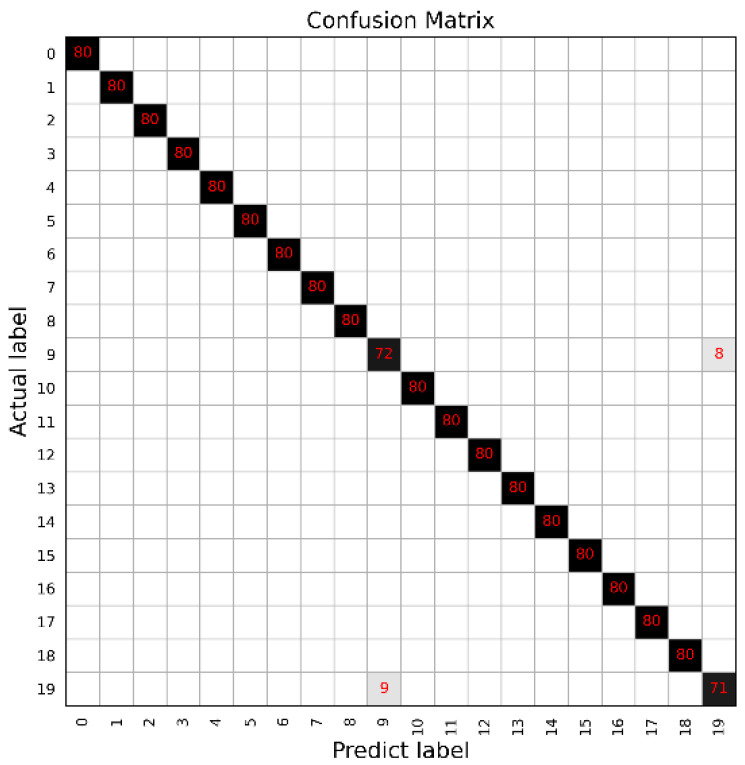
The confusion matrix of datasets A and B (20 healthy conditions) using optimal signals with our CNN architecture.

However, an accuracy of 100% is difficult to achieve in practical engineering as the number of fault categories is far more than 10. To study the classification performance of the proposed method for more fault categories, the datasets A, B, C and D presented in Table 2 were combined in pairs to increase the health conditions to 20. The new datasets, which include 20 categories of bearing signals, were further processed by the proposed method, and the classification accuracies of the testing data are shown in Table 5. An accuracy of more than 96.9% was achieved. As comparisons, the raw signals, which were not processed by the multiparameter adjusting Duffing system before classification, were also processed using the proposed CNN and the traditional CNN; the optimal signals were also processed using the traditional CNN. The classification accuracies of the testing data are shown in Table 5. One can see that the classification accuracies are enhanced by either pre-processing the raw signals or using our CNN architecture, and the classifications accuracies achieved the highest when both methods were adopted. Therefore, both the optimization method and our CNN architecture play important roles in enhancing classification accuracies, showing that the proposed method has the capability to realize high-performance fault classification and feature extraction results better than conventional methods. It is noted that as the accuracy is different for every training and inference, each classification accuracy presented in Table 5 was obtained by taking the average of the accuracy results of five simulations. To observe which healthy conditions were difficult to classify, Figure 9 shows the confusion matrix of datasets A and B using optimal signals with our CNN architecture. Only two normal signals under different loads had misclassification, which means that two normal signals contain similar information and features and it was difficult to classify them correctly.

#### 4.2.4. Influence of the Population Number on Classification Accuracies and Calculation Time

In addition to the classification accuracies, the calculation time is another important index to evaluate the performance of a classification method. Both indexes are affected by the population number, which is studied in the subsection.

**Figure 10:**
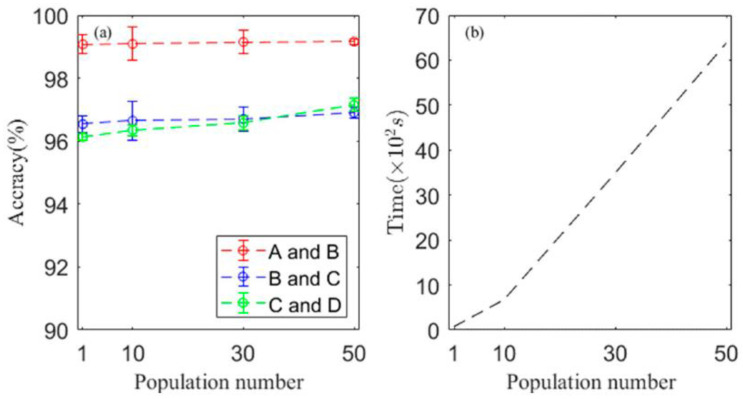
(**a**) The classification accuracies and (**b**) the calculation time against the population number.

The datasets shown in Table 4 were re-processed using the proposed method with different population numbers. The accuracies and calculation time against the population number are shown in Figure 10. It can be seen from Figure 10a that when the population number increased from 1 to 50, the classification accuracies had slight increases of less than 1%, meaning that the population number had a relatively low influence on the classification accuracies. Figure 10b shows that increasing the population number significantly increases the calculation time. When the population number increased from 1 to 50, the cost time for the optimization process increased from 30 s to 6500 s. Therefore, it is possible to get an acceptable high-accuracy classification result in a short time using the proposed method.

#### 4.2.5. Visualizations of Feature Maps and Networks

Generally, CNN is an efficient tool to extract features, but it is hard to understand how CNN processes grey images. In this subsection, the feature maps and networks are plotted for a better understanding of the powerful feature extraction and classification capacities of CNN.

**Figure 11:**
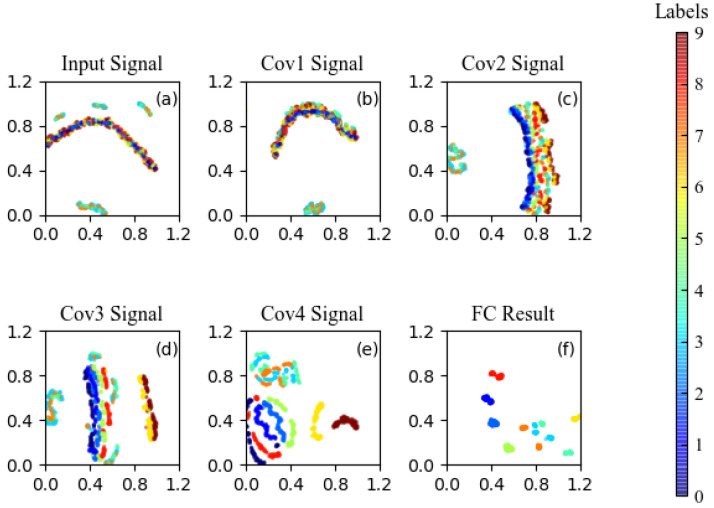
Feature visualization via t-SNE: feature representations of (**a**) input images, (**b**–**e**) images obtained through four convolutional layers, and (**f**) images obtained through the last fully-connected layer.

For dataset A, Figure 11 shows the feature distributions of some representative layers in the CNN visualized by the t-distributed stochastic neighbor embedding (t-SNE) [48]. The features of the input signals of the CNN, which are the output signals of the Duffing system, are not obvious. The features are continuedly separated by each convolutional layer, and the classification result of each fault type becomes obvious after the fourth convolutional layer. Moreover, the features in the fully connected layer are easier to be divided and an accuracy of 100% can be obtained.

## 5. Conclusions

A novel intelligent fault diagnosis method combining the adaptive multiparameter stochastic resonance (SR) as a weak-fault feature extraction method and the normal convolutional neural network (CNN) as a fault classification method is proposed in this paper for bearing fault enhancement diagnosis. A multiparameter bistable Duffing system that can realize SR for large-parameter signals is introduced as a weak-fault feature extraction model, whose optimal output signal-to-noise ratio (SNR) can be achieved adaptively using a proposed hybrid optimization algorithm (HOA) combining the genetic algorithm (GA) and simulated annealing (SA) with high optimization speed. Therefore, poor-quality raw data can be pre-processed by the adaptive multiparameter-adjusting SR of the Duffing system. A mapping method that can convert the SR outputs of the Duffing system into grey images is proposed. The obtained grey images are further processed by a normal CNN with batch normalization (BN) and dropout layers, thus achieving fault enhancement classification. The research shows that a relatively high output SNR can be obtained from the adaptive multiparameter-adjusting Duffing system using the proposed HOA that has a better optimization performance compared to the GA. Therefore, weak features of raw data can be significantly enhanced. The bearing fault dataset from the CWRU bearing data center was pre-processed by the feature extraction method and was further processed by the proposed CNN model. Classification accuracies of 100% were achieved for 10 categories of bearing signals and those of more than 96.9% were achieved for 20 categories of bearing signals, which is better than other methods. The influences of the population number on the classification accuracies and calculation time were further studied, indicating that the proposed method can realize a rather good classification result in a short time. The feature map and network visualization are presented by t-SNE to show how the features were identified by the CNN. Hence, it was demonstrated that the proposed method can realize high-performance fault enhancement classification and identification. Moreover, to make full use of the advantage of SR in detecting weak signals, the abundant information of SR output, such as the frequency domain output, can be further combined with other appropriate intelligent methods for fault classification or prediction in future work.

## Figures and Tables

**Table 1:** Drive end bearing details and fault frequency.

Position on Rig	Inside Diameter (inch)	Outside Diameter (inch)	Thickness (inch)	Ball Diameter (inch)	Defect Frequencies (Multiple of Running Speed in Hz)		
					Inner Ring	Outer Ring	Rolling Element
					${f_{\mathrm{BPFI}}}^{\prime }$	${f_{\mathrm{BPFO}}}^{\prime }$	${f_{\mathrm{BPFP}}}^{\prime }$
Drive end	0.9843	2.0472	0.5906	0.3126	5.4152	3.5848	4.7135

**Table 2:** Details of the bearing datasets.

		Inner Race			Outer Race			Ball			Normal
Category labels		0	1	2	3	4	5	6	7	8	9
Fault diameters (inches)		0.007	0.014	0.021	0.007	0.014	0.021	0.007	0.014	0.021	0
Dataset A:Load 1	Train	320	320	320	320	320	320	320	320	320	320
	Test	80	80	80	80	80	80	80	80	80	80
Dataset B:Load 2	Train	320	320	320	320	320	320	320	320	320	320
	Test	80	80	80	80	80	80	80	80	80	80
Dataset C:Load 3	Train	320	320	320	320	320	320	320	320	320	320
	Test	80	80	80	80	80	80	80	80	80	80
Dataset D:Load 4	Train	320	320	320	320	320	320	320	320	320	320
	Test	80	80	80	80	80	80	80	80	80	80

**Table 3:** Optimization ranges of each adjustable parameter of dataset A.

Fault Position	Fault Diameter (inch)	Searching Range of Each Parameter			
		*k*	*a*	*b*	*ε*
inner race	0.007	[0.1,15]	[0.1,10]	[0.1,10]	[0.1, 5]
	0.014				[0.1, 5]
	0.021				[0.15, 10]
ball	0.007				[0.5, 15]
	0.014				
	0.021				
outer race	0.007				[0.15, 10]
	0.014				[0.1, 10]
	0.021				[0.1, 5]

**Table 4:** Accuracies for the signals with 10 conditions using optimization method.

Dataset	A	B	C	D
Accuracy	100%	100%	100%	100%

**Table 5:** Accuracies for the signals with 20 healthy conditions using three different classification methods.

	Optimal Signals with Our CNN Architecture	Optimal Signals with Traditional CNN Architecture (without BN and Dropout Layers)	Raw Signals with Our CNN Architecture	Raw Signals with Traditional CNN Architecture (without BN and Dropout Layers)
Datasets A and B	99.16%	98.06%	98.44%	94.31%
Datasets B and C	96.90%	95.94%	95.44%	85.44%
Datasets C and D	97.15%	95.96%	95.94%	90.44%
